# Surgical and Pathological Characteristics of Papillary Thyroid Cancer in Children and Adolescents

**DOI:** 10.1155/2012/125389

**Published:** 2011-11-17

**Authors:** Davor Dzepina

**Affiliations:** Department of ENT—Head and Neck Surgery, Sisters of Charity University Hospital, Vinogradska 29, 10000 Zagreb, Croatia

## Abstract

*Background*. Thyroid carcinoma is a relatively rare pediatric pathology, comprising around 3% of all childhood tumors. We investigated parameters of tumor aggressiveness, multicentricity, and locoregional metastatic spread patterns in patients up to 18 years of age and made comparison with the older group. All patients were operated upon with total thyroidectomy, with or without lymph-node neck dissection. *Results*. Patients with papillary carcinoma present with more advanced stage, larger primary tumor, and more commonly present with palpable thyroid and/or neck node. Overall, papillary cancer demonstrated pathological aggressiveness as defined by our criteria in 60%, multicentricity in 40%, and locoregional metastatic foci in 77% of cases. Multicentric tumor foci in both thyroid lobes and tumor aggressiveness were identified as a risk factor for metastatic development. *Conclusion*. By observing clinicopathological parameters, we demonstrated that papillary thyroid cancer behaves more aggressively in the younger group. We recommend total thyroidectomy with careful intraoperative exploration of thyroid bed and lateral neck in search for possible metastatic spread. In case of positive findings, it is obligatory to perform a standard neck dissection, keeping in mind that neck lymphonodes are primary site of locoregional recurrence. With meticulous attention to technical aspects of operation, perioperative morbidity should be minimal.

## 1. Introduction

It is generally accepted that papillary thyroid carcinoma in children and adolescent population has a different clinical presentation and course than in adults [1]. Initial diagnostic presentation in pediatric patients tends to be in more advanced stage, namely, the larger primary tumor, the higher incidence of primary tumor multicentricity and higher incidence of locoregional metastatic spread. More specifically, palpable neck lymphonodes are most commonly presenting sites in children (up to 90%). In addition, significant primary tumor aggressiveness has been demonstrated in young age [1]. It is already demonstrated that primary thyroid tumor aggressiveness (as measured by nuclear atypia, tumor necrosis, and lymphovascular invasion) correlates with metastatic disease, independently from tumor size [2]. Consequently, histological grade should be set as prognostically important component and included in any classification system of differentiated thyroid cancer [3]. 

Despite these aggressive characteristics, specific prognosis in pediatric age is better than in adults. As a consequence, several recommendations have been made that propose less aggressive therapeutic approach (less than total thyroidectomy and no neck dissection). On the contrary, some researchers advise more aggressive therapy, including total thyroidectomy, neck lymphonode dissection, and postoperative radioiodine ablation [4–6]. However, recurrent disease and locoregional spread still present major clinical concern regarding optimal extent of the initial surgical therapy, in pediatric population specifically. Several major studies attempted to evaluate influence of patient and tumor characteristics, as well as treatment factors, but without clear recommendations regarding possible risk factors for developing recurrent disease [7–11].

 In this paper, we show our retrospective clinical material in 16 patients up to 18 years of age, who underwent surgical therapy at our institution for diagnosis of thyroid papillary carcinoma. The aim of the presentation is to give insight into clinical and selected pathological characteristics (tumor size and aggressiveness, multicentricity, and locoregional spread) of the disease in pediatric age, assess their role in cancer risk, and identify possible critical parameters for developing metastatic disease. 

## 2. Material and Methods

This study is a retrospective analysis of data from 16 pediatric patients with papillary thyroid cancer, ages from 10–18. All patients were operated upon at the Department of ENT—Head and Neck Surgery, University Hospital “Sisters of Charity”, Zagreb, Croatia during the 28-year period (1980–2008). Patients' data are summarized in Table 1. 

All patients underwent total thyroidectomy with or without neck dissection (paratracheal or some type of lateral neck dissection). We used Robbins et al.'s neck dissection classification data from Consensus Statement on the Classification and Terminology of Neck Dissection [12]. Medical data were collected from patient documentation (intraoperative reports, recurrences, FNAB results, and additional thyroid diagnosis) and final pathology reports, as well as from the Hospital Registry for Thyroid Diseases were reviewed. Postoperatively, we followed *Diagnostic and Therapeutic Guidelines for Differentiated Thyroid Cancer*, issued by Croatian Thyroid Society; postoperative diagnostic scintigraphy was performed with 1–3 mCi ^131^I. High-risk patients were put to 100–200 mCi ^131^I ablation without L-T4. Posttherapeutic whole body scintigraphy was performed 5–8 days after ^131^I. Six to twelve months later, the patients were followed up with the exploration of neck ultrasound, FT4, TSH, Tg, and TgA (without L-T4) measurements and afterwards yearly. 

All patients were assigned to groups for variables of age (≤18 and comparison group >18 years), gender, size (diameter) and pathological parameters of primary tumor aggressiveness, multicentricity, type of neck dissection performed, and presence of locoregional metastatic foci in neck. Grading of pathological aggressiveness, multicentricity, and locoregional spread is demonstrated in Table 2. We applied statistical analysis of differences with *χ* ^2^ test and model of multivariate logistic regression for risk factors for metastatic locoregional spread. Statistical significance level was set at *P* ≤ 0.05.

## 3. Results

From the total of 699 cases with papillary thyroid cancers of all ages, 16 patients (2.3%) with age up to 18 years fulfilled inclusion criteria, 13 females, and 3 males. The average age at the moment of diagnosis was 16, with a range of 10 to 18 years. Mean tumor size was 2.2 cm. Majority of patients presented with palpable thyroid mass, 12/16 or 75%, and palpable neck node in 8/16, or 50%. There were only 4 microcarcinoma cases (25%), which is in sharp contrast to older population (45% microcarcinoma). Distribution of parameters of pathological aggressiveness, multicentricity, neck dissection, and metastatic spread and comparison by age group (≤18 and >18 years) are shown in Table 3. Results showed that 60% of pediatric tumors were aggressive, according to our chosen criteria. Forty percent of tumors were multicentric, with foci in both lobes twice as often as in the single lobe (13% versus 27%). Total thyroidectomy was the operation of choice in all cases. Neck dissection was performed in 76.5% patients (35% paratracheal, 41% lateral). There were 77% positive metastatic tumors: paratracheal metastases in 23% and lateral neck metastases in 54%. Bilateral metastatic neck involvement was demonstrated in 50% of cases. 

Overall, no age differences were found for pathological aggressiveness (*P* = 0.19) and multicentricity (*P* = 0.89) even though younger group had significantly wider aggressiveness (34% versus 16%). Younger patients had significantly more neck dissections performed (*P* = 0.005) and more metastatic spread as well (*P* = 0.000). There were no cases of postoperative recurrent nerve paralysis on postoperative indirect laryngoscopy. We had two cases of temporary early postoperative hypocalcaemia, which were successfully controlled by calcium carbonate and Rocaltrol p. o. There was one case of permanent hypocalcaemia (recurrent case).

There were two cases of disease recurrence, both locoregional: one patient with single neck recurrence (lateral neck metastatic foci after initial TT and paratracheal ND) and one patient with two recurrences (both in lateral neck regions after initial TT and paratracheal ND). Both recurrences were detected on follow up by neck palpation and ultrasonography with cytology, and pathologic Tg; both cases were amenable to surgical therapy and were put to further radioiodine treatment. No patients with N0 neck developed recurrence. In all patients, postoperative and postablative average follow up was at least 5 years, and they were alive and free of disease. Multivariate analysis revealed the presence of multicentric foci in *contralateral lobe* and higher tumor aggressiveness (group III) to be an independent risk factor for regional metastatic spread (OR 3.119 and 2.591, resp.). Male gender was identified as an additional risk factor (OR 1.919), while older age was associated with lower risk for metastatic development (OR 0.537). Lymphomatous goiter bears lower risk (OR 0.633) (Table 4).

## 4. Discussion

Different potential prognostic factors of papillary thyroid cancer have been revealed so far, most importantly patient age, tumor grade, and extension (extrathyroid invasion, distant metastasis, and less frequently regional). Papillary cancer characteristics and behavior in children and adolescent population is subject of several controversies, mainly about the most appropriate surgical therapy, the use of postoperative iodine ablation and TSH suppression, as well as follow-up modalities [1, 13]. It is well demonstrated that clinical course of this disease in children and adolescent population is significantly different: the worse initial clinical presentation in children and, paradoxically, the better disease prognosis than in adult population [14, 15]. Children tend to present with larger primary tumors, greater incidence of neck lymphonode metastasis, and distant metastasis as well [16]. This presentation/prognosis discrepancy is not yet fully understood. Possible explanations are more common occurrence of well-differentiated forms of tumor and more effective response to postoperative TSH suppression with thyroid hormone [1].

In our series, we demonstrated aggressive characteristics of papillary cancer in 60% of cases. Most aggressive cases came in the form of a wider tumor aggressiveness, that is, thyroid capsule invasion, penetration of adjacent thyroid tissues, and/or perivascular/perineural spread. Additionally, mean tumor size in younger age group was significantly larger than in adult population. The role of tumor size was commented recently in the review of Sherman, who emphasized that smaller size of thyroid gland in children can lead to earlier thyroid and extrathyroid spread of disease [17].

Papillary thyroid cancer multicentricity is a well-described feature of this tumor, with estimated frequency range from 22% to 49% [1]. There are major disagreements about the importance of papillary cancer multicentricity; its etiology and clinical significance are not yet fully understood [6, 18–20]. As a consequence, many authors propose total thyroidectomy as an adequate surgical approach, claiming that more extensive operation decreases the likelihood of recurrence [21]. There are other different reports as well. Our study revealed that 40% of papillary carcinomas developed multicentric foci, with most of them appearing synchronously in both thyroid lobes. Thus, multicentricity, when it occurred, was bilateral in more than two-thirds of multicentric cases. Further, on multivariate analysis multicentricity was found to be the most important risk factor for development of metastatic spread (OR 3.119). Interpretations of these findings strongly signify the need for an implementation of more aggressive surgery of the primary tumor, incorporating complete removal of the contralateral thyroid lobe in the same act as thyroidectomy.

Positive neck lymphonodes are a frequent presenting sign in children/adolescent population. Locoregional metastatic spread is identified as one of the most important risk factors preceding distant dissemination. Neck metastases occur in 15%–60% and with meticulous search can be found in up to 90% of cases [22, 23]. However, even for distant spread, chances of long-term survival are significant, particularly in younger age group [24]. Half of all our cases had palpable neck node on presentation. On final pathology, there were 77% positive metastatic tumors: paratracheal metastases in 23%, and lateral neck metastases in 54%. Bilateral metastatic neck involvement was demonstrated in 50% of cases. It has been demonstrated that metastatic disease, locoregional and distant, seems to be most important prognostic factor for the good response to therapy [25]. Data from present study clearly demonstrate the significance of detailed preoperative neck examination and careful intraoperative exploration, with special emphasis on lateral neck regions.

Presently, total or less commonly subtotal thyroidectomy is considered optimal surgical therapy for papillary thyroid cancer in all preoperatively diagnosed cases [18, 26]. Conducted studies showed that more extensive operation (total thyroidectomy) usually leads to significant reduction of recurrence [9, 13, 27]. A more conservative surgical approach (lobectomy) is advocated by some authors as well, emphasizing less risk of surgical and postoperative morbidity (temporary or permanent hypoparathyroidism and recurrent laryngeal nerve injury) [11, 28, 29]. 

Thyroid cancer recurrence is more common in extremes of age, most notably in patients less than 20 years of age, as well as older than 60 [4, 30]. Generally, studies performed on all age populations revealed higher chances for recurrence in procedures including less than total thyroidectomy. This higher aggressiveness of thyroid cancer demonstrated in younger population does not necessarily bear higher risk for fatal outcome, which is more common in older population [14, 15]. Our both recurrence cases had a large primary tumor in the thyroid gland; one had aggressive characteristics (thyroid capsule invasion and perivascular, perilymphatic spread), and both had initially neck metastatic disease. 

Regarding intraoperative and early postoperative complications rate, in our material, we have not found significant differences in perioperative complication rates in children versus older patients, with extent of surgery being the same as for adults. We had two cases of temporary and one case of permanent hypocalcaemia. In recurrent cases, reoperative surgery, which consisted of lateral neck dissection, was performed without any complications. However, in this cohort of patients, we advise that strong emphasis should be put on minimizing morbidity related issues, bearing in mind excellent long-term disease prognosis. 

In conclusion, in this study, we investigated parameters of papillary thyroid cancer aggressiveness, multicentricity, and locoregional metastatic spread in children/adolescent population. Overall, papillary thyroid cancer demonstrated aggressiveness in 60%, intraglandular dissemination in 40%, and metastasized locoregionally in 77% of cases. By observing clinicopathological parameters and their distribution across selected groups, we demonstrated that papillary cancer behaves more aggressively in younger age group. Multicentric foci in both thyroid lobes and wider tumor aggressiveness were identified as a risk factor for metastatic development. We support the need for total thyroidectomy and meticulous intraoperative exploration of the thyroid bed and lateral neck, with surgical extirpation of all potential microscopic disease foci. For positive regional metastatic disease, standard paratracheal and/or modified radical neck dissection is obligatory part of surgery, in the same act as total thyroidectomy, keeping in mind that neck lymphonodes are primary sites of locoregional recurrence. With meticulous attention to technical aspects of surgery, perioperative morbidity of parathyroid glands and recurrent laryngeal nerve should be minimal.

## Figures and Tables

**Table 1:** Epidemiologic, clinical, and pathological data of 16 children with papillary thyroid carcinoma, operated upon with total thyroidectomy w/o neck dissection.

Case no.	Gender	Age (years)	Tm size (cm)	Thyroid gland multicentricity	Aggresivenesss^1^	Neck dissection^2^	Metastatic spread^3^	Recurrence no.	Additional dx
(1)	F	10	2,7	Contralateral lobe	1	2	2	0	—
(2)	F	13	0,9	—	0	1	0	0	—
(3)	F	13	5	Contralateral lobe	2	2	2	1	—
(4)	F	15	1,1	—	0	0	0	0	—
(5)	F	15	2,8	—	0	1	1	2	—
(6)	F	15	1,2	—	1	1	0	0	—
(7)	M	16	1,9	Contralateral lobe	1	2	2	0	—
(8)	F	16	2,3	—	2	2	2	0	—
(9)	F	17	0,4	—	0	1	0	0	Lymphomatous goiter
(10)	F	18	2,1	—	1	1	1	0	—
(11)	F	18	0,5	—	0	1	1	0	Lymphomatous goiter
(12)	F	18	1	Ipsilateral lobe	0	0	0	0	Lymphomatous goiter
(13)	M	18	5,5	Contralateral lobe	1	2	2	0	—
(14)	M	18	2	Contralateral lobe	2	2	2	0	—
(15)	F	18	2,6	Ipsilateral lobe	2	2	2	0	—
(16)	F	18	3	—	2	0	0	0	—

^1^Aggresivity: (1) Sharply demarcated; encapsulated tumor; (2) No clear tumor border; tumor capsule invasion; (3) Thyroid capsule invasion, perivascular, perineural spread, penetration of adjacent soft tissues, fat tissue, muscle or cartilage invasion.

^2^Neck dissection (ND): (1) Not done; (2) Paratracheal ND; (3) Paratracheal and lateral ND.

^3^Metastatic spread: (1) No metastatic spread; (2) Paratracheal region involved; (3) Paratracheal and lateral regions involved.

**Table 2:** Study variables: gradation by severity of pathohistological features of tumor aggressiveness, intraglandular dissemination, and locoregional metastatic spread.

Gradation	Variables			
	Pathological aggressiveness of primary tumor	Intraglandular dissemination (multicentricity)	Neck dissection*	Locoregional metastatic spread
Grade I	Sharply demarcated; encapsulated	No multicentric foci	Not done	No metastatic spread
Grade II	No clear tumor border; tumor capsule invasion	Ipsilateral lobe spread	Paratracheal	Paratracheal
Grade III	Perivascular, perineural spread; penetration of adjacent soft tissues; fat tissue, muscle, cartilage invasion	Bilateral/Contralateral lobe spread	Paratracheal and Lateral	Paratracheal and Lateral

*Neck dissection classification from: Robbins et al. [12].

**Table 3:** Comparison of chosen pathological parameters by age groups (≤18 years, ≥18 years).

Characteristic	Age ≤ 18 y, *n* = 16 (2,3%)	Age ≥ 18 y, *n* = 683 (97,7%)	*P*
Pathologic aggressiveness	9/15 (60%)	302/651 (46,4%)	*P* = 0,19
Capsule invasion/no clear border	4 (26%)	198 (30,4%)	
Wider aggression	5 (34%)	104 (16%)	

Multicentricity	6/15 (40%)	222/645 (34,4%)	*P* = 0,89
Ipsilateral lobe	2 (13%)	81 (12,5%)	
Bilateral/contralateral lobe	4 (27%)	141 (21,9%)	

Neck dissection	13/17 (76,5%)	300/674 (44,5%)	*P* = 0,005
Paratracheal	6 (35,3%)	200 (29,7%)	
Paratracheal and lateral	7 (41,2%)	100 (14,8%)	

Metastatic spread	10/13 (77%)	180/671 (26,8%)	*P* = 0,0000
Paratracheal	3 (23%)	92 (13,7%)	
Paratracheal and lateral	7 (54%)	88 (13,1%)	

**Table 4:** Risk factors for regional metastatic spread, a logistic regression model.

	OR	95% CL		*P*
		Upper	Lower	
Gender				
Male	1,919	1,30	2,84	0,012
Female	0,591	0,35	0,77	0,012
Older Age	0,537	0,38	0,75	0,0003
Multicentricity				
Ipsilateral	1,062	0,63	1,78	0,8187
Bilateral/contralateral	3,119	2,12	4,59	0,0000
Expansive tm growth				
No expansion	0,540	0,38	0,77	0,0007
Capsule invasion/no clear border	1,022	0,71	1,47	0,9056
Wider aggression	2,591	1,69	3,97	0,0000
Additional diagnosis				
Lymphomatous goiter	0,633	0,40	1,00	0,0485
